# The first report of rabies in stone marten (*Martes foina*) in Chaharmahal and Bakhtiari Province (Iran)

**DOI:** 10.1002/vms3.1328

**Published:** 2023-11-21

**Authors:** Seyed Hossein Zamzam, Mohammadreza Ghorani, Fahime Eslami, Saeed Mostofi

**Affiliations:** ^1^ General Department of Veterinary Medicine of East Azerbaijan Province Tabriz Iran; ^2^ Department of Pathobiology Faculty of Veterinary Medicine University of Tabriz Tabriz Iran; ^3^ Chaharmahal and Bakhtiari Provincial Office of Department of Environment Shahrekord Iran

**Keywords:** FAT, Iran, rabies, stone marten, zoonosis disease

## Abstract

Rabies is a prevalent endemic and zoonotic fatal disease, which is normally transmitted to humans by contact (scratches and bites) from infected animals. The present paper deals with the first documented evidence of rabies in the stone marten (*Martes foina*). Rabies symptoms were observed in a marten in Chaharmahal and Bakhtiari province. The animal with a strange demeanour approached people without fear, which was died after some while. Samples were taken from its brain (cerebellum, hippocampus, and hypothalamus), shortly after death. In this report, laboratory evidence of rabies by fluorescent antibody (FAT) was proved. The present work is valuable because of the environmental importance of the stone marten. Hence, sensitive surveillance and advanced reporting systems should be regularly monitored on suspected cases of rabies in animals and humans to control and prevent this deadly disease. This involves exposure history, clinical examinations, symptoms and experimental results. Rabies can be controlled by fast diagnostic tests and vaccination.

## Graphical abstract

The stone marten with clinical signs of rabies was identified. Sampling was done from it. The samples were sent for further laboratory tests. The Florescent Antibody Technique (FAT) confirmed the presence of the rabies virus. In this article, we reported the first case of rabies in Stone Marten in Iran. The importance of this case report is that, first, stone martens are endangered animals and, second, because they live far away from humans, reports of rabies in stone martens are rare.

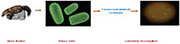

## 1 INTRODUCTION

Rabies is a deadly disease‐causing preventable death, most commonly in Africa and Asia, with effectual postexposure prevention for humans, negligible animal control and vaccination plans. Rabies is first found in wild animal hosts in developed countries where it is directly transmitted to humans from wild animals like bats, skunks, raccoons and foxes, rather than domestic animals (Blanton et al., 2011; Kilic et al., 2006).

Rabies is still an ecumenical threat in contrast to extensive pet vaccination programs. Generally, As the disease is fatal, it is essential to consider rabies postexposure prophylaxis (RPEP) for all victims who were bitten unreasonably by cats and dogs. A 10‐day quarantine is suggested for ferrets, dogs and cats. Victim prevention may not be essential when utilising proper animal quarantine. However, it is essential to identify the first clinical symbols of the disease in the quarantined animal (Ruskin et al., 1993).

When the animal is a runaway dog or cat, local rabies epidemiology should be regarded to initiate PREP. Humans can be potentially at risk of rabies from the bite of any wild animal based on the rabies transmission level in local wildlife populations. In case it is not possible to test the rabies in wild animals, immediate RPEP should be taken (Goldstein, 1994).

It is important to take the other two cases of tetanus infection and local wound infection, and rabies in the bite of pets or wild animals, while not ignoring prevention. The infection risk is greater in all deep puncture wounds from dog bites, cat, human bites, or monkey, hand or leg wounds, any bites in immunosuppressed patients, and injury during surgical repair. Moreover, prophylactic antibiotics should be used to treat these wounds. Attacks on humans are rare by wild animals. According to studies, there are three main causes for wolf attacks indicating that the attack may be introduced by humans, the human may be considered prey, and it may be a rabid wolf (Linnell et al., 2002). Humans, foxes and wolves rarely interact with each other. Nevertheless, tragic events occasionally occur. As long as wild animal and mammalian attacks are uncontrolled in developing countries, rabies is an important public health problem (Türkmen et al., 2012).

Domestic animals contamination is abundant in Iran as rabies is endemic in wildlife (Esfandiari et al., 2010). Rabies needs to be suspected strongly by unexpected attacks by wild animals. Hence, domestic animals must be vaccinated to prevent rabies infection and transmission to humans. Wild animals need to be avoided, particularly animals behaving abnormally, and bats must be kept away from buildings and public houses. In this paper, the first occurrence of rabies was reported in stone marten in Chaharmahal and Bakhtiari (Iran).

## 2 MATERIALS AND METHODS

### 2.1 Case history

2.1

On 27 April 2022, a stone marten died at the site of the Koohrang area with geographic coordinates with the scientific name *Martes foina*. It is in the Least Concern (LC) class in the International Union for Conservation of Nature (IUCN) (419818‐3590941). A few days before the death, symptoms of not being afraid of humans and getting closer to humans were observed in this animal, and reported by local people.

### 2.2 Sampling

2.2

**FIGURE 1:**
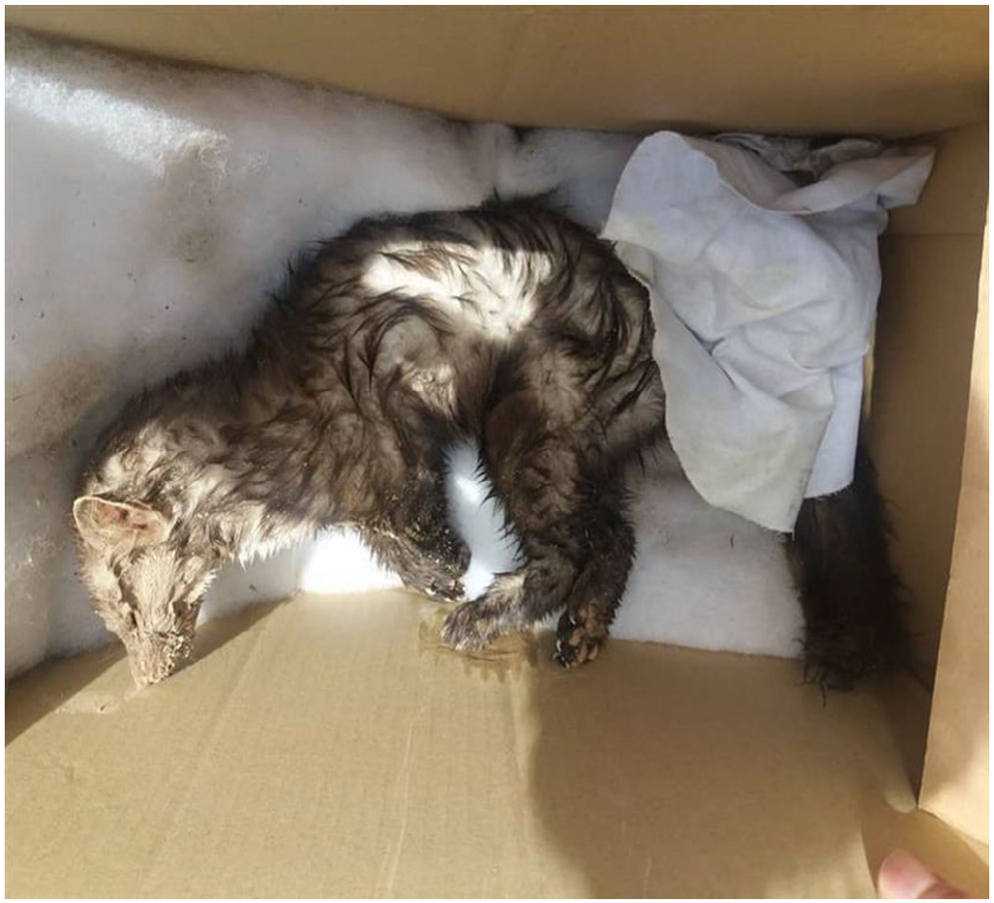
The death rabid stone marten.

The brain samples (hippocampus, hypothalamus and cerebellum) were obtained from the stone marten immediately after death (Figure 1). The samples were taken under aseptic circumstances, and in full obedience with safety principles. The fresh specimens were sent to the laboratory on ice.

### 2.3 Laboratory investigations

#### 2.1 2.3.1 FAT

Using FAT, brain tissue samples were examined for rabies infection. FAT was used on fresh samples based on WHO and OIE (International Office of Epizootics, Biological Standards Commission, International Office of Epizootics, & International Committee, 2008; Dean, 1996) with the Iran Veterinary Organization.

## 3 RESULTS

**FIGURE 2:**
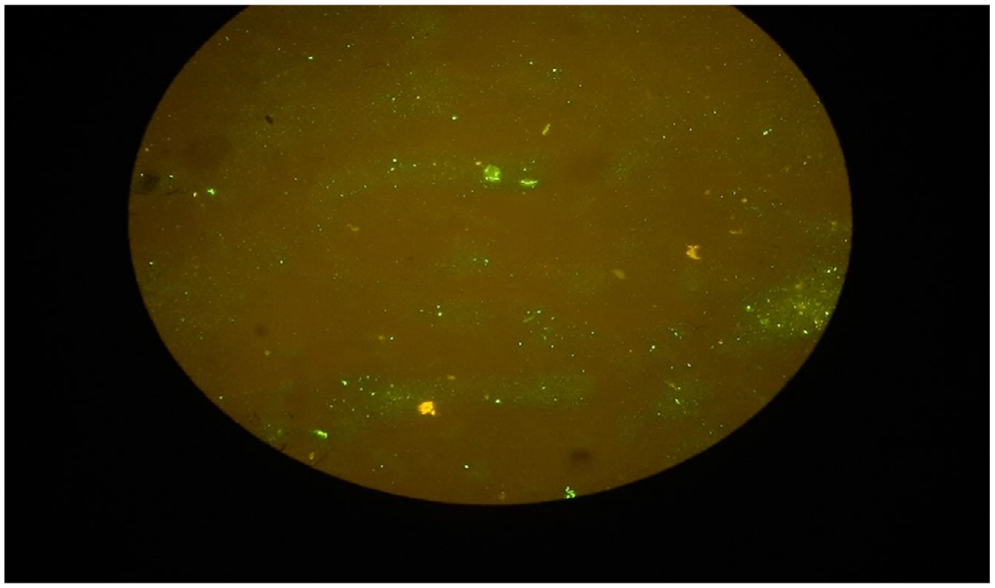
The FAT result of samples taken from stone marten.

FAT was considered to confirm rabies in the samples (Figure 2).

## 4 DISCUSSION

Rabies is an acute zoonotic viral disease it causes encephalitis in mammals (Lafon, 2005; Rupprecht et al., 2010). Assessing rabies reservoirs indicated that foxes, jackals and dogs are the most prevalent rabies repositories in northern Iran. Wolves are still dominant in the western and northwestern areas (Simani, 2003). There are different animal species affected by rabies in different areas. On the Caspian shores, the main rabid animals are stray jackals and dogs, while foxes and wolves have a more significant impress in the mountainous area of the central plateau (Bokaei et al., 2009). Considerable seasonal changes were found in another reportage of animal rabies in Kerman province from 1993 to 2003, in some suspected and approved rabid animals, and its peak was in fall and autumn. The only risk of rabies virus transmission is a rabid animal bite, and wolves, Chinese badgers and domestic dogs are the primary sources of rabies in Asia (James & Dubovi, 2016). Ghorani et al. reported rabies in a grey wolf (*Canis lupus*) in Chaharmahal and Bakhtiari province and a red fox (*Vulpes vulpes*) in southwest Iran (Ghorani & Eslami, 2022; Ghorani et al., 2022). In the current work, stone marten strangely behaved and approached humans with no fear. Considering the oral vaccination strategy of foxes, the stone marten case is the first explanation of rabies in a land species after seven years of absence in Saxony‐Anhalt (Stöhr et al., 1994). In August 2001, a stone marten (*Martes foina*) suspected of rabies was found in the city of Burg (Saxony‐Anhalt, Germany), and rabies was confirmed by the regional veterinary laboratory by rabies tissue culture inoculation test and mouse inoculation (Müller et al., 2004).

**Highlights**

- In this article, we reported the first case of rabies in Stone Marten in Iran.
- The epidemiological aspects of rabies were reviewed.
- The importance of this case report is that, first, stone martens are endangered animals and, second, because they live far away from humans, reports of rabies in stone martens are rare.

No definite clinical manifestations of rabies exist most importantly. Moreover, all cases of behavioural changes should be taken as a differential etiological diagnosis in rabies until definitive elimination. To immunise humans and animals against rabies, recombinant, inactivated vaccines and live attenuated were developed. Enzootic dog rabies is still a severe problem in most Asian, African and Latin American countries, with considerable mortality in humans and domestic animals. Various doses of rabies vaccine are used in these countries. Thus, comprehensive rabies control programs are essential, though they are not low‐cost to operate and maintain. The possibility of human bites was increased by the entry of wild animals into cities in cold seasons.

Rabies control in enzootic areas is also complicated by the attendance of cats and wild canids in Africa along with various species of wildlife resources in the Caribbean islands. Rabies control agencies in North America and Europe, work with public support in the following regions: first, eliminating stray cats and dogs and controlling the pet's movement (quarantine is rarely used in epizootic situations); second, immunisation of dogs and cats with appropriate vaccines to break the virus transmission chain; third, the institution of control programs and rabies prevention in wildlife indicates important regional reservoir host(s) of animals; fourth, experimental detections to achieve precise incidence information and approve clinical observations; fifth, measurement of the all‐control measures’ successfulness by monitoring; and sixth, guarantees cooperation with public education programs (James & Dubovi, 2016).

## 5 CONCLUSION

A sensitive surveillance system is required to control and prevent this deadly disease, and to track suspected rabies cases completely in humans and animals through an advanced reporting system. Such a system includes exposure history, clinical examinations, signs and laboratory results. About public and zoonotic health, fast diagnosis of rabies is essential. Vaccination of wildlife animals is also required in endemic countries to control the disease, which is less considered.

## AUTHOR CONTRIBUTIONS

**Seyed Hossein Zamzam**: Writing—original draft preparation. **Mohammadreza Ghorani**: Writing—reviewing; editing and conceptualisation. **Fahimeh Eslami**: Sampling. **Saeed Mostofi**: Laboratory investigations.

## CONFLICT OF INTEREST STATEMENT

The authors declare that there is no conflict of interest.

### ETHICS STATEMENT

The authors confirm that the ethical policies of the journal, as noted on the journal's author guidelines page, have been adhered to. No ethical approval was required as this is a review article with no original research data.

### PEER REVIEW

The peer review history for this article is available at https://publons.com/publon/10.1002/vms3.1328.

## Data Availability

The data that support the findings of this study are available from the corresponding author upon reasonable request.
